# Postoperative Analgesia in Children- Comparative Study between Caudal Bupivacaine and Bupivacaine plus Tramadol

**Published:** 2009-08

**Authors:** Meena Doda, Sambrita Mukherjee

**Affiliations:** 1 Specialist & HOD, Dept.of Anaesthesiology, ESIC Hospital, Noida, India- 201301; 2 Anaesthesiologist, Dept.of Anaesthesiology, ESIC Hospital, Noida, India- 201301

**Keywords:** Caudal analgesia, Bupivacaine, Tramadol

## Abstract

**Summary:**

Thirty children, ASAI-II, aged between 2yrs-5yrs, undergoing sub umbilical operation (inguinal and penile surgery) were selected for this double blind study. They were randomly divided in two groups, group A and group B. Group A(n=15) received 0.25%bupivacaine 0.5ml.kg^−1^ and Group B (n=15) received 0.25% bupivaeaine 0.5ml.kg^−1^ and tramadol 2mg.kg^−1^ as single shot caudal block. Postoperative pain was assessed by a modified TPPPS (Toddler-Preschool Postoperative Pain Scale) and analgesic given only when the score was more than 3. In the first 24 hrs it was observed that the mean duration of time interval between the caudal block and first dose of analgesic was significantly long(9.1hrs) in Group B as compared to Group A (6.3hrs) which was much shorter(p<0.01). There was no significant haemodynamie changes, motor weakness or respiratory depression in both groups. This study concluded that addition of tramadol 2mg.kg^−1^ to caudal 0.25% bupivacaine 0.5ml.kg^−1^ significantly prolong the duration of postoperative analgesia in children without producing much adverse effects.

## Introduction

The society of Paediatric Anaesthesia^1^, on it's 15^th^ annual meeting at New Orleans, Louisiana (2001) clearly defined the alleviation of pain as a “basic human right”, irrespective of age, medical condition, treatment, primary service response for the patient care or medical institution. Finely et al^2^ observed that many types of so called “minor” surgery (e.g. circumcision) can cause significant pain in children.

The goal of post operative pain relief is to reduce or eliminate pain with minimum side-effects and in our setup as cheaply as possible. Effective pain relief means a smooth postoperative period, increased patient compliance and an early discharge from hospital. Langlade et al^3^ suggested that the postoperative pain treatment must be included in the anaesthetic planning even before induction of anaesthesia, adopting the idea of ‘managing pain before it occurs’.

Over the years various regional anaesthetic procedures has gained popularity for postoperative analgesia because in addition to providing effective postoperative pain relief, they also reduce the requirement of general anaesthesia intraoperatively without significant side-effects and maintaining a smooth intra and postoperative period. Caudal block has proved useful in a variety of subumbilical operations^4^ in children for providing both intra operative and post operative analgesia. Objective of present study was to compare the quality and duration of analgesia, after a single shot caudal block with bupivacaine alone and bupivacaine plus tramadol, and thereby try to find out whether tramadol can be an effective adjuvant to bupivacaine for providing postoperative analgesia in children undergoing subumbilical surgeries.

## Methods

After obtaining institutional approval and parental written informed consent, thirty children aged between 2-5yrs, weighing between 10-18 Kg and of ASA I and II physiologic status were enrolled for the study. These patients were scheduled for sub-umbilical surgeries like herniotomy and penile surgery under general anaesthesia by a single surgeon. The patients were randomly allocated in two groups.

Group A received single shot caudal block with 0.25% bupivacaine 0.5ml.kg^−1^ and Group B received 0.25% bupivacaine 0. 5ml.kg^−1^ plus tramadol 2mg.kg^−1^, after induction of anaesthesia. Any children having allergy to bupivacaine or any contraindication to neuraxial blockade were excluded from the study.

The patients were induced with halothane and 50% nitrous oxide in oxygen inhalation via face mask. Intravenous cannulation was done using 22G cannula, then atropine 0.02mg.kg^−1^, ondansetron 0.1mg.kg^−1^ and midazolam 0.1mg.kg^−1^ were given i.v as premedication. After induction, caudal block was then given in right lateral position by a 22G needle under aseptic condition. Syringes containing an equal volume of either 0.25% bupivacaine 0.5m1.kg^1^ or 0.25% bupivacaine 0.5ml.kg^−1^ plus tramadol 2mg.kg^−1^ were prepared and given to the investigator who was blinded to the identity of drug(s). He gave the caudal blocks. Then the surgery was continued under inhalational anaesthesia via mask. Intraoperative heart rate, respiratory rate, blood pressure (NIBP) and oxygen saturation (SpO2) was monitored. After recovery from general anaesthesia the patient was shifted to PACU and his vitals and pain was assessed by a 10-point TPPS score^5^ (Table 1) by a blinded investigator. The child's motor power, any side-effects and sedation score(0= Eyes open, 1= Eyes open to speech, 2=Eyes open when shaken, 3= unrousable) was also noted. Assessment was done every 5-min for the first 30-min, then every 15-min for next 1hr, then hrly for next 2 hrs and then at 4, 6, 8, 10, 14, 18 and 24hr by the same blinded investigator.

**Table 1:** Pain assessment method in children(TPSS Score*)

Variable	Score 0	Score 1	Score 2
Verbal complaint/cry	None	Once Only	>once
Groan/Moan/Grunt	None	Once only	>once Facial
Expression	Neutral	One Grimace	Grimace>1
Restless Motor behaviour	None	One episode	>one episode
Rub/ touch painful area	None	Once only	>Once

* According to Toddler-Preschool Postoperative Pain Scale

## Data Processing

ANOVA with multiple comparisons was used for comparisons between the groups. Using Chi squared (X^2^) test compared the non-parametric data. *p*<0.05 was regarded as statistically significant.

## Results

The two groups were comparable in age, weight and duration of surgery (Table 2).

**Table 2:** Patient data and duration of anaesthesia

Variables	Group A(n=15)	Group B(n=15)
Age(yrs)	2.7±1.6	3.6±1.34
Weight(kg)	11.3±3.77	12.3±4.8
Gender(M:F)	15:0	15:0
Duration of anaesthesia(min)	32.2±8.75	29.8±8.09
Baseline heart rate(per min)	103±9.15	94±10.33

P<0.05

While comparing the quality of postoperative analgesia between the two groups it was seen that the Group A started having mild pain after 3hrs and the pain was significant after 6hrs whereas in Group B the child was pain free for almost 5 hrs and started having significant pain after 8 hrs which needed analgesic supplementation with syrup Paracetamol at the dose of 10 mg.kg^−1^. Significant pain is described as one that has a pain score of more than 3 (Table 3).

**Table 3:** Average time interval between caudal analgesia and first dose of analgesic

Patient group	Mean duration(hrs±sd)
Group A (n=15)	6.3±2.93
Group B (n=15)	9.1±3.14

*p*<0.05

**Fig 1:**
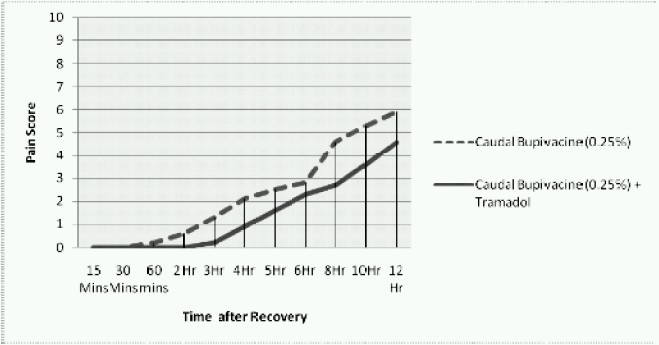
Changes in pain score along with time

When pain score was plotted against time in a graph, it was seen that the score was 0 upto 2 hrs and then started to increase and reached a score of 3 only after 9 hrs in Group B, where as in Group A the pain score started to attain 3 after 6 hrs.(Fig 1).

It was also seen that the children in Group A needed more doses of paracetamol syrup in first 24 hrs than Group B (Table 4).

**Table 4:** Number of doses of paracetamol syrup given to both groups in first 24 hrs

No of doses of Paracetamol	Group A	Group B
1	0	0
2	1	11
3	6	3
4	8	1

p<0.05

The vitals of patients in both groups remain stable during operation and the incidences of emergence agitation were much less in both groups rather than the patients undergoing surgery under general anaesthesia without caudal block.

There was no major difference in sedation score between the two groups after recovery. 13.3% patients in Group A and 6.6% in Group B developed motor weakness. It was also observed that incidences of postoperative urinary retention was 20% in Group A and 13.3% in Group B. Nausea and vomiting was slightly more in Group B (26.67%) than Group A(20%), (Table 5).

**Table 5:** Incidences of adverse effects in two groups(n)

Incidences of adverse effects	Group A (n=15)	Group B (n=15)
Motor Weakness	2	1
Urinary Retention	3	2
Nausea & Vomiting	3	4

*p*=not significant

## Discussion

Ease of performance and reliability makes caudal block the most commonly performed block in children. Caudal administration of bupivacaine is a widespread regional anaesthetic technique for intra- and postoperative analgesia during lower limb, anoperineal, penoscrotal and abdominal surgical procedures in children^6^‐^8^. Tramadol is a centrally acting opioid analgesic, used for treating moderate to severe pain. It is a synthetic agent, made of racemic mixture of two enantiomers- (+) tramadol and (-) tramadol and it appears to have actions at the μ-opioid receptor as well as the noradrenergic and serotonergic systems^9^. Tramadol was developed by the German pharmaceutical company Grünenthal GmbH in the late 1970s and marketed under the trade name Tramal. As an analgesic it's equipotent to meperidine without any respiratory depressant action. The most commonly reported adverse drug reactions are nausea, vomiting, sweating and constipation. Drowsiness is reported, although it is less of an issue than for opioids.

In our study, we found that by adding tramadol 2mg.kg^−1^ to caudal bupivacaine (0.025%) 0.5ml.kg^−1^ in children undergoing sub-umbilical operation, significantly increased the duration of pain free period post-operatively. Similar results were reported by Gune et al 10 during a study of children undergoing hypospadias repair showed that caudaltramadol provides better and longer lasting postoperative analgesia than i.v tramadol. Senel et al 11 in a study on children undergoing herniorrhaphy showed that, caudal administration of bupivacaine with the addition of tramadol resulted in superior analgesia with a longer period without demand for additional analgesics compared with caudal bupivacaine and tramadol alone without an increase of side effects. The incidence of emergence agitation, which is frequently seen during recovery from inhalational anaesthesia in children, were much less in children with preoperative caudal block in both groups and it was more less in Group B and this is supported by a previous study of Weldon et al^12^ who reported that effective postoperative analgesia may reduce the incidence of emergence agitation with sevoflurane anaesthesia. The degree of sedation was comparable in two groups. The potency of single shot caudal bupivacaine was increased by addition of tramadol because in our set up it was neither technically possible nor cost effective to use caudal epidural catheter and maintain postoperative analgesia with bupivacaine alone. A prolong and effective postoperative analgesia to children means a cooperative child with less emotional and haemodynamic stress and rapid recovery with less hospital stay. Mean duration of postoperative analgesia with caudal bupivacaine was 6.3 hrs whereas with addition of tramadol it increased up to 9.1 hrs, without increasing the dose as well as the side effects of bupivacaine as it was shown in various studies^13^,^14^. A higher dose of tramadol could have caused nausea and vomiting whereas increasing the dose of bupivacaine could have caused more motor weakness and urinary retention.^15^

Our study concluded that caudal administration of tramadol 2mg.kg^−1^ along with 0.25% bupivacaine 0.5ml.kg^−1^ significantly increased the duration and quality of postoperative analgesia in children undergoing sub-umbilical operation, without producing significant adverse effects.
